# Survival from brain tumours in England and Wales up to 2001

**DOI:** 10.1038/sj.bjc.6604604

**Published:** 2008-09-23

**Authors:** S C Short

**Affiliations:** 1 Department of Oncology, University College Hospital, Euston Road, London NW1 2PG, UK

It will come as no surprise to most clinicians involved in the treatment of primary brain tumours that there is little evidence of improvement in outcome between the late 1980s and the late 1990s. The majority of these tumours are gliomas and in this group most are high-grade tumours, which are notorious for having resisted the therapeutic endeavours that have improved outcome in so many other cancers. It is only in the last few years that new insights into the biology of gliomas and significant changes in the treatment of these tumours has occurred, which we predict will alter the outcome in the coming decade.

High-grade glial tumours typically present with a short history of focal neurological deficit, which progresses over days to weeks and may mimic a stroke-like illness. In most cases, a space-occupying lesion can be demonstrated on CT or MRI scanning and the diagnosis is confirmed by biopsy and/or resection, which is always subtotal because of the infiltrating nature of the disease. The much wider use of high-quality CT and MRI scans in patients presenting in this way during the late 1980s and 1990s accounts for some of the increased incidence of brain tumours reported during this time. During the same period, the classification of tumour subtypes was clarified in a new WHO classification in which glioblastoma was formally grouped with astrocytic tumours, but no major changes in disease definition occurred (Kleihues *et al*, 1993; Louis *et al*, 2007).

In Europe, the standard approach to management of these tumours, which has persisted until very recently has been maximal surgery followed by external beam radiotherapy. The influence of the extent of surgery has never been addressed in a randomised study although many series have suggested that it is a prognostic indicator (Wrensch *et al*, 2006). It is likely that improvements in surgical technique, particularly the use of stereotactic biopsy also contributed to more frequent diagnosis of tumour during this time period, but made little impact on outcome. During this time, the development of radiotherapy technology also meant that more patients were treated using CT-based techniques to improve definition and verification of the tumour target as well as radiation dosimetry. However, because of the apparent inherent radioresistance of these tumours these advances did not impact significantly on outcomes (Oppitz *et al*, 1999; Chan *et al*, 2002).

Against this background, the observed increase in incidence but lack of improvement in survival is not surprising. The fact that the overall survival (OS) actually worsened is probably because of increased diagnosis in patient groups that carry the worst prognosis, particularly the elderly and those with a poor performance status. The reversal of the deprivation gap is also most easily explained, as the authors suggest, by differences in access to imaging and diagnostic services so that the more affluent groups were more likely to be correctly diagnosed with a tumour, but in circumstances in which their prognosis remained very poor.

The rather depressing statistics presented in this paper make it clear that improvements in diagnosis and in technical aspects of treatment that occurred during the late 1980s and 1990s were insufficient to improve the outcome for brain tumour patients. More recently, however, the approach to diagnosis and treatment of these tumours has changed and there is now optimism that OS is beginning to improve in some tumour types. Advances in molecular techniques have allowed the definition of tumour subtypes that respond differently to treatment. Most significantly, it has been recognised that some glial tumours with specific chromosome abnormalities, particularly oligodendrogliomas with loss of 1p19q, respond favourably to chemotherapy and radiotherapy and represent a significantly better prognostic group (Cairncross *et al*, 2006; van den Bent *et al*, 2006). This has prompted ongoing investigation into the relevance of other genetic markers in gliomas and considerable effort is going in to identifying genomic profiles that may be useful as predictive or prognostic indicators (Dehais *et al*, 2006).

The most significant advance in treatment of glioma has been the demonstration that in grade IV tumours (glioblastoma), the addition of concomitant and adjuvant chemotherapy with temozolomide to postoperative radiotherapy can improve OS (Stupp *et al*, 2005). In this randomised study, patients allocated to temozolomide given continuously during radiotherapy and for an additional 6 months thereafter had median survival of 14.6 months compared with 12.1 months in the group treated with radiotherapy only and 2-year survivorship was increased from 11 to 26%. In a parallel translational study, the activity of the DNA repair enzyme MGMT was measured in tumour tissue. These data suggested that response to temozolomide may depend on the activity of this enzyme, as patients with methylation of the promoter region for the gene and therefore reduced activity levels, showed more benefit from the addition of temozolomide (Hegi *et al*, 2005). Ongoing studies will address the utility of this approach in other tumour types and contribute further to our understanding of the impact of tumour genotype and epigenetics on response to specific treatments. In parallel with the successful application of molecularly targeted agents in other tumours, the role of drugs that can target specifically upregulated growth factor pathways are also being explored in gliomas (Mellinghoff *et al*, 2005). This will define a more individualised treatment approach that will complement ongoing developments in brain imaging, neurosurgical and radiotherapy technology.

The development of new individualised approaches to treating these tumours will mean that coordination between neuropathologists and the multidisciplinary team responsible for managing these patients will become essential for treatment optimisation. It will also provide an impetus to include more of these patients in well-designed clinical studies that can further define the role of specific interventions in well-defined tumour subgroups.
